# Extreme enthalpy‒entropy compensation in the dimerization of small solutes in aqueous solution

**DOI:** 10.1007/s00249-024-01722-y

**Published:** 2024-10-15

**Authors:** David J. Scott, Donald J. Winzor

**Affiliations:** 1 School of Biosciences, University of Nottingham, Sutton Bonington, LE12 5RD UK; 2 Research Complex at Harwell, Rutherford Appleton Laboratory, Oxfordshire, OC11 OFA UK; 3 School of Chemistry and Molecular Biosciences, University of Queensland, Brisbane, QLD 4072 Australia

**Keywords:** Enthalpy‒entropy compensation, Small solute dimerization, Urea, Aliphatic carboxylic acids, N-methylformamide

## Abstract

This communication summarizes findings from the earliest encounters with extreme enthalpy‒entropy compensation, a phenomenon first detected in the 1950s by a reappraisal of isopiestic and calorimetric measurements on aqueous urea solutions in terms of solute self-association. Because concurrent studies of carboxylic acid association were confined to measurement of the equilibrium constant by conductance, IR spectrophotometry or potentiometric titration measurements, temperature-independence of the dimerization constant was mistakenly taken to signify a value of zero for Δ$H^o$ instead of (Δ$H^o$ ‒ TΔ$S^o$). In those studies of small-solute self-association the extreme enthalpy‒entropy compensation was reflecting the action of water as a reactant whose hydroxyl groups were competing for the solute carbonyl involved in self-association. Such action gives rise to a positive temperature dependence of Δ$H^o$ that could well be operating in concert with that responsible for the commonly observed negative dependence for protein‒ligand interactions exhibiting extreme enthalpy‒entropy compensation, where the solvent contribution to the energetics reflects changes in the extent of ordered water structure in hydrophobic environments.

## Introduction

Observations of extreme enthalpy‒entropy compensation in interactions of proteins with charged ligands (Anusiem et al. 1968; Waksman et al. 1993; Sleigh et al. 1999; Dragan et al. 2004) as well as nonpolar counterparts (Kilpatrick et al. 1986; Krishnamurthy et al. 2006; Lafont et al. 2007) gave rise to expressions of amazement as well as disbelief. Considerations of such findings to be remarkable (Gilli et al. 1994) and a paradox (Krishnamurthy et al. 2006) have led to their dismissal as statistical artifacts (Sharp 2001; Cornish-Bowden 2002; Chodera and Mobley 2013). More constructive interpretations of the findings in terms of an experimental phenomenon have entailed the concomitant existence of compensatory conformational changes in protein stuucture (Williams et al. 2004; Frederick et al. 2007; Edwards et al. 2009; Ferranti and Gorski 2012) and/or changes in water structure (Lumry and Rajender 1970; Clothia 1974; Reynolds et al. 1974; Lafont et al. 2007; Breiten et al 2013; Fox et al. 2018). Any detailed rationalization of enthalpy‒entropy compensation in protein‒ligand systems clearly requires satisfactory account to be taken of both of these phenomena (Privalov and Crane-Robinson 2017; Dragan et al. 2017; Fox et al. 2018; Scott et al. 2019). In that regard an obvious attraction of the latter explanation is that any gain in the enthalpic contribution ($\Delta H^o$) to the standard free energy $(\Delta G^o )$ from enhanced hydrogen bonding involving water hydroxyls is necessarily accompanied by a concomitant loss in entropy contribution ($T\Delta S^o$) because of decreased randomness of a more ordered water structure.

More definitive evidence of solvent involvement as a potential source of enthalpy‒entropy compensation should emanate from studies of the dimerization of small solutes in aqueous solution, where the solute contribution to compensatory entropy change is essentially confined to the loss in randomness stemming from the relative immobilization of two solute monomers in dimer formation. Indeed, the existence of extreme enthalpy‒entropy compensation was first reported in the 1950s (Schellman 1955) for the dimerization of urea. The present retrospective appraisal of those and other results for small-solute systems exposes further shortcomings of the traditional inherent assumption that water may be regarded as an inert solvent—a situation encountered in studies of the association of aliphatic carboxylic acids (MacDougall and Blumer 1933; MacInnes and Shedlovsky 1932; Saxton and Darken 1940; Katchalsky et al. 1951; Klotz and Franzen 1962; Schrier et al.1964).

We begin this investigation with the early studies of urea dimerization (Scatchard et al. 1938; Gucker and Pickard 1940), where consideration has been given to the energetics of the whole thermodynamic system (Schellman 1955).

## The dimerization of urea

**Fig. 1:**
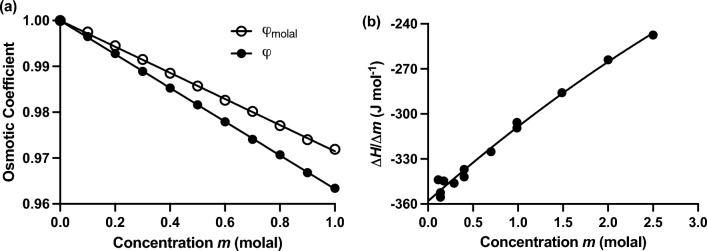
Anonalous thermodynamic behavior of urea solutions at 25 °C. **a** Negative deviations from Raoult’s Law revealed by concentration dependence of the osmotic coefficient for solvent (φ) derived from isopiestic measurements. ●, Results from Table II of Scatchard et al. (1938) with φ expressed in terms of mole-fraction; ○, Corresponding dependence with the osmotic coefficient a molal quantity (Winzor and Wills 2019). **b** Negative concentration dependence of the heat of dilution for aqueous solutions of urea:. [Data taken from Fig. 1 of Gucker and Pickard (1940).]

The anomalous thermodynamic behaviur of aqueous urea solutions first came to light in isopiestic measurements at 25 °C of the chemical potential of water in sucrose, glycerol and urea solutions (Scatchard et al. 1938). Whereas those measurements of the osmotic coefficient for solvent (*φ*) on solutes such as glycerol and sucrose exhibited the positive deviations from Raoult’s Law that we now recognize to be consistent with interpretation of thermodynamic nonideality on the statistical-mechanical basis of excluded volume (McMillan and Mayer 1945; Winzor and Wills 1995) or molecular crowding (Minton 1983), the corresponding departure from thermodynamic ideality was negative for urea (●, Fig. 1a). This evidence of negative deviations from Raoult’s Law for aqueous urea solutions was confirmed by concurrent estimation of the osmotic coefficient from freezing point depression measurements (Chadwell and Politi 1938). Those findings were soon followed by a calorimetric study (Gucker and Pickard 1940) that also revealed unusual solution behavior for aqueous urea solutions in that the heat of dilution was negative (Fig. 1b) rather than the positive prediction for dipolar substances (Scatchard and Kirkwood 1932).

### The Schellman (1955) interpretation

The reported negative heats of dilution (Fig. 1b) were taken to signify the operation of short-range enthalpic interactions; and attributed to intermolecular hydrogen bonding between carbonyl and amino groups of urea molecules. The thermodynamic consequences of this situation were illustrated for a model involving indefinite self-association in which the same association equilibrium constant *K* governed the stepwise addition of monomer to form dimer, trimer, tetramer, etc.—a model now referred to as isodesmic indefinite self association (Van Holde and Rossetti 1967). Under thermodynamically ideal conditions the total molal concentration $m$ is related to its monomer counterpart $m_1$ by the expression

1 $$ m = \frac{m_1 }{{(1 - Km_1 )}} $$

On the other hand, the total molality is also given by the stoichiometric relationship

2 $$ m = m_1 + 2m_2 + 3m_3 + \cdots $$

which, on the incorporation of Eq. (1), can be written in the form

3 $$ m = \frac{m_1 }{{(1 - Km_1 )^2 }} $$

**Fig. 2:**
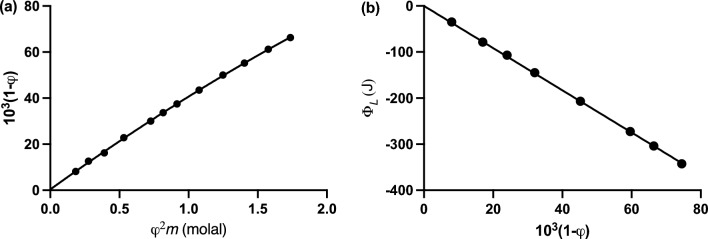
Schellman (1955) interpretation of the anomalous behavior in terms of urea self-association. **a** Analysis of the *φ‒m* dependence (Fig. 1a) by the application of Eq. (13) to obtain *K* (taken as the dimerization constant) from the slope. **b** Evaluation of Δ$H^o$ from the heat of dilution data (Fig. 1b) by its analysis according to Eq. (14). [Data taken from Figs. 1 and 2, respectively of Schellman (1955).]

These expressions were then applied to the isopiestic measurements for urea (Scatchard et al. 1938), where the osmotic coefficient was defined in terms of solvent thermodynamic activity $$a_s $$ as

4 $$ \varphi = - \frac{\ln a_s }{{(M_s /1000)m}} $$

in which $$M_s , $$ the molecular weight of solvent (water) is divided by 1000 to conform with the definition of molality as moles of solute per kg of solvent. Under the presumed condition of thermodynamic ideality the relationship between $$a_s$$ and the partition coefficient then becomes

5 $$ \varphi = - \frac{{\ln \left( {1 - \sum \chi_i } \right)}}{(M_s /1000)m} \approx \frac{\sum \chi_i }{{(M_s /1000)m}} \approx \frac{(M_s /1000)\sum m_i }{{(M_s /1000)m}} \approx \frac{\sum m_i }{m} $$

where $\chi_i$ is the mole-fraction of species *i*, the concentration scale employed by Scatchard et al. (1938) in the measurement of the partition coefficient *φ*. Combination of Eqs. (1), (2) and (5) then leads to the relationship

6 $$ \left( {1 - \varphi } \right) = Km\varphi^2 $$

which allows the association constant *K* to be determined from the limiting slope of the dependence of $(1 - \varphi )$ upon $\varphi^2 m$ **(**Fig. 2a**).** A value of 0.041 molal^‒1^ was thereby obtained (Schellman 1955). This estimate of *K* signifies a standard free energy change, $\Delta G^o$, of +7.9 kJ/mol.

A similar strategy was adopted (Schellman 1955) in the interpretation of the anomalous (negative) values of the relative heat of dilution for aqueous urea solutions reported by Gucker and Pickard (1940). Provided that all association steps are characterized by the same standard enthalpy change $\Delta H^o$, the relative heat capacity $\Phi_L$ is related to the osmotic coefficient φ by the expression (Schellman 1955)

7 $$ \Phi_L = \Delta H^o (1 - \varphi ) $$

where vales of $\Phi_L$ were obtained from the empirical relationship (now expressed in J/mol)

8 $$ \Phi_L = - {359}.{5}m + {28}.{53}m^2 - 0.1913m^3 $$

reported by Gucker and Pickard (1940). From the slope of the essentially linear dependence (Fig. 2b) of $\Phi_{L }$ upon $(1 - \varphi )$, Schellman (1955) obtained an estimate of ‒8.8 kJ/mol for $\Delta H^o$. Combination of this value of the standard enthalpy change with that of $\Delta G^o { }$ in the Gibbs–Helmholtz equation then yielded an entropic contribution (*T*Δ$S^o$) of ‒16.7 kJ/mol to the energetics of the system. Although Schellman (1955) made no comment about the size of the entropic contribution, his thermodynamic interpretation of the energetics of urea dimerization provided the first reported example of extensive enthalpy‒entropy compensation in an aqueous solute solution.

### More rigorous confirmation of the Schellman findings

The analysis of isopiestic measurements has been amended subsequently (Winzor and Wills 2019) by taking into account the fact that the solvent thermodynamic activity, traditionally expressed in terms of mole-fraction *χ* [Eq. (5)], is a molal parameter because of the constraints (constant temperature and pressure) under which the thermodynamic measurements are made (Hill 1959, 1968). Although such substitution of $\sum {\chi_i } m$ for the effective solute molality in the calculation of the partition coefficient *φ* is consistent with the condition of thermodynamic ideality assumed by Schellman in his analysis of the isopiestic measurements on aqueous urea solutions (Sctchard et al. 1938), some consideration of the consequences of thermodynamic nonideality is required over the large solute concentration range covered in those experiments.

In measurements of the osmotic coefficient *φ* by the isopiestic procedure the magnitude of the solvent chemical potential in the vapor phase ($\mu_s$) is established by including in each experiment a solution of a solute for which the molal concentration dependence of *φ* is known. That value of the solvent chemical potential also applies to solutions of the solute of interest (urea in the present case) because of their coexistence in partition equilibrium with the same vapor phase. Under the operative constraints of constant temperature (*T*) and pressure (*P*) that pertain in isopiestic measurements the solvent chemical potential, $(\mu_s )_{T,P}$, is described in terms of its standard state value, $(\mu_s^o )_{T,P}$, by the expression (Winzor and Wills 1995)

9 $$ \frac{{(\mu_s^o )_{T,P} - (\mu_s )_{T,P} }}{RTM_s m} = (1 + C_2 m + \cdots ) = \varphi_{{\text{molal}}} $$

where the molal concentration of solute, $$m = n/(n_s M_s )$$, is the ratio of the number of solute molecules ($$n)$$ present in a mass $$n_s M_s$$ of solvent; and where the molal second virial coefficient. ($$C_2$$) is related to the molal thermodynamic activity of solute, $$a, $$ by the expression (Winzor and Wills 2019)

10 $$ a = m {\text{exp}}(2C_2 m + \cdots ) $$

Because the partition coefficient (Fig. 1a) was originally defined with solute concentration measured on the mole-fraction scale, Eq. (5), its magnitude was recalculated (○, Fig. 1a) to obtain the required dependence of $\varphi_{{\text{molal}}}$ upon $m$ [see also Fig. 1 of Winzor and Wills (2019) and the discussion thereof for further details of this process].

Under conditions of thermodynamic ideality for solute self-association, the thermodynamic activity $a$ can be written as

11 $$ a = m_1 + m_2 + \cdots $$

where truncation of the summation at dimer seems justified on the basis of the small magnitude (0. 041 molal^‒1^) reported (Schellman 1955) for the dimerization constant.

The corresponding expression for total urea concentration ($$m)$$ is then

12 $$ m = m_1 + 2m_2 + \cdots $$

**Fig. 3:**
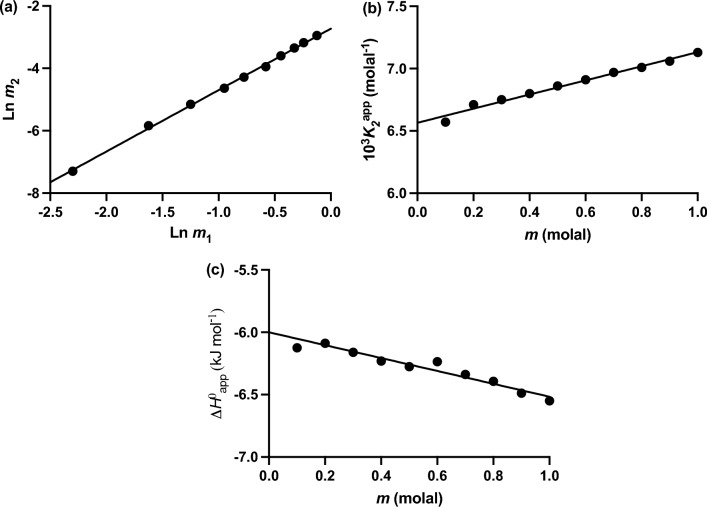
Use of the $\varphi_{molal} - m$ dependence (Fig. 1a) and heat of dilution data (Fig. 1b) for more rigorous thermodynamic characterization of urea dimerization. **a** Check on the stoichiometry by means of the logarithmic form of the law of mass action for solute self-association. **b** Extrapolation of apparent dimerization constants to obtain the thermodynamic dimerization constant $K_2 .$ **c** Evaluation of Δ$H^o$ by extrapolating apparent values obtained from Eq. (20) to zero solute concentration. [Data taken from Figs. 2 and 3 of Winzor and Wills (2019).]

On the grounds that $m_2 = (m - a){\text{ and }}m_1 = \left( {m - 2m_2 } \right) = \left( {{\text{a}} - m_2 } \right)$ the validity of truncating urea self-association at dimer over the concentration range 0 ‒ 1 molal was verified by the slope (2.033 ± 0.005) of the plot of results in accordance with the logarithmic form of the law of mass action for a monomer‒dimer equilibrium (Fig. 3a). Calculation of the apparent dimerization constant as $K_2^{{\text{app}}} = m_2 /m_1^2$ for each experimental point leads to the concentration dependence of $K_2^{{\text{app}}}$ shown in Fig. 3b, and an estimate of 0.0659 (± 0.0005) molal^‒1^ for $K_2$ from the ordinate intercept.

Calculation of the standard enthalpy change Δ$H^o$ for dimerization was based on the reasoning adopted by Schellman (1955) except that Eq. (7) was rearranged as

13 $$ \Delta H_{{\text{app}}}^o = \frac{\Phi_L }{{1 - \varphi_{{\text{molal}}} }} = \frac{\Phi_L m}{{m - a}} = \frac{\Phi_L m}{{m_2 }} $$

where Eq. (8) was again used to obtain $\Phi_L$, and where the standard enthalpy change is denoted as an apparent value because of assumed thermodynamic ideality in the derivation of Eq. (13). Extrapolation of those estimates of $\Delta H_{{\text{app}}}^o$ for each urea concentration to zero solute concentration to eliminate the effects of thermodynamic nonideality is shown in Fig. 3c, from which an estimate of ‒6.04 (± 0.05) kJ/mol for $\Delta H^o$ is obtained (Winzor and Wills 2019). Its combination with the standard free energy change $\Delta G^o$ of 6.74 (± 0.02) inferred from the above estimate of $K_2$ in the Gibbs‒Helmholtz equation yields an estimate of ‒12.78 (± 0.07) kJ mol for the entropic contribution ($T\Delta S^o$) to the energetics of dimerization at 25 °C. Although this more rigorous interpretation of the energetics of the system has yielded different values for the three energy parameters, it substantiates the Schellman (1955) observation of extensive enthalpy‒entropy compensation that gives rise to the small positive value for $\Delta G^o$ for urea dimerization—an enthalpically driven equilibrium reaction.

## The dimerization of aliphatic carboxylic acids

**Fig. 4:**
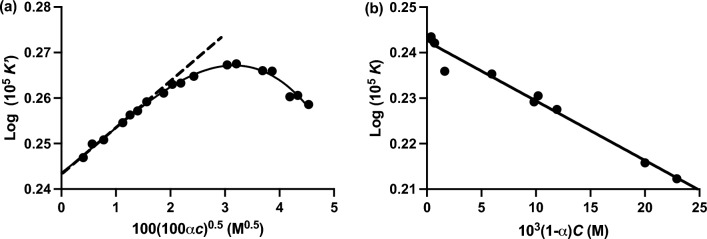
Anomalous ionization behaviour of aqueous acetic acid solutions at 25 °C. **a** Plot of the concentration dependence of the classical ionization constant *K’* in accordance with Eq. (15) to obtain the thermodynamic ionization constant *K* from the limiting slope. [Data taken from Table II of MacInnes and Shedlovsky (1932).] **b** Application of Eq. (16) to the anomalous data to obtain a quantitative description in terms of the empirical parameter B. [Data taken from Table II of Saxton and Darken (1940).]

Thermodynamic evidence of reversible self-association in aqueous solutions of acetic acid emanated from vapor-pressure measurements at 25 °C (MacDougall and Blumer 1933). At the same time attempts were being made to obtain an empirical description of the anomalous ionization behavior of aqueous carboxylic acid solutions revealed by conductance measurements under the same conditions (MacInnes and Shedlovsky 1932; Saxton and Darken 1940). That description of the ionization behavior of aqueous acetic acid at 25 °C (MacInness and Shedlovsky 1932) in terms of the classical ionization constant $K^{\prime}$ for the simplest ionization reaction,

14 $$ {\text{HA}}{{ \leftrightarrows }}{\text{H}}^+ + {\text{A}}^- ;\quad K^{\prime} = C_{H^+ } C_{A^- } /C_{HA} = \alpha^2 C/(1 - \alpha ) $$

with *α* the apparent degree of ionization ($C_{A^- }$ as a fraction of total molar concentration $C$) is shown in Fig. 4a. The corresponding concentration dependence of the thermodynamic ionization constant $K$ for electrolytes is then (Debye and Hückel 1923)

15 $$ \log K = \log K^{\prime} - 1.013\sqrt {C} $$

Conformity of results with Eq. (15) is evident from the slope at low concentrations in Fig. 4a, which presents their analysis of conductance data for aqueous acetic acid solutions [Table II, Fig. 2 of MacInnes and Shedlovsky (1932)]. Furthermore, the subsequent deviation from that dependence finds quantitative description in terms of the empirical relationship (Saxton and Darken 1940)

16 $$ \log K^{\prime} = \log K - B(1 - \alpha C) $$

with $B = 0.14$ (Fig. 4b). This empirical parameter has been shown to be a quantitative measure of the dimerization association constant (Katchalsky et al. 1951).

### Thermodynamic interpretation in terms of dimerization

A thermodynamic interpretation of the anomalous ionization of carboxylic acids utilizes the assumption that the cyclic dimer (MacDougall and Blumer 1933) undergoes negligible ionization because of the involvement of the hydroxyl groups in hydrogen bonding with carbonyl groups. The solution composition is then governed by the monomer‒dimer equilibrium

17 $$ {\text{2HA}}{{ \leftrightarrows }}({\text{HA}})_2 { }\,\,\,K_2 = C_{(HA)_2 } /C_{HA}^2 $$

as well as that for monomer ionization [Eq. (14)]. Because the expression for total carboxylic acid is then

18 $$ C = C_{A^- } + C_{HA} + 2C_{(HA)_2 } = C_{A^- } + C_{HA} \left( {1 + 2K_2 C_{HA} } \right) $$

that for the classical ionization constant becomes

19 $$ K^{\prime} = \frac{\alpha^2 C}{{\left( {1 - \alpha } \right)[1 + 2K_2 \left( {1 - \alpha C} \right)]}} \approx \left[ {\frac{\alpha^2 C}{{(1 - \alpha )}}} \right][1 - 2 K_2 \left( {1 - \alpha C} \right)] $$

On the basis that $\ln (1 - x) \approx - x$ for small *x*, the base 10 logorithmic transform of Eq. (18) may be written as

20 $$ \log K^{\prime} = \log K - 2(0.4343)K_2 (1 - \alpha C) $$

where log K refers to the thermodynamic ionization constant incorporating the Debye‒Hückel factor [Eq. (15)]. A theoretical expression, $$B = 0.8686K_2 , $$ has thus been derived (Katchalsky et al. 1951) for the constant in Eq. (16), the empirical analysis of the ionization behavior of aqueous carboxylic acid solutions deduced by Saxton and Darken (1940). Substitution of the empirically obtained estimate of 0.14 for *B* (Fig. 4b) yields a dimerization constant $$K_2$$ of 0.16 $$M^{ - 1}$$ that is in reasonable agreement with the reported value of 0.185 $$M^{ - 1}$$ obtained by thermodynamic analysis of vapor pressure measurements on aqueous acetic acid solutions under the same conditions (MacDougall and Blumer 1933).

**Fig. 5:**
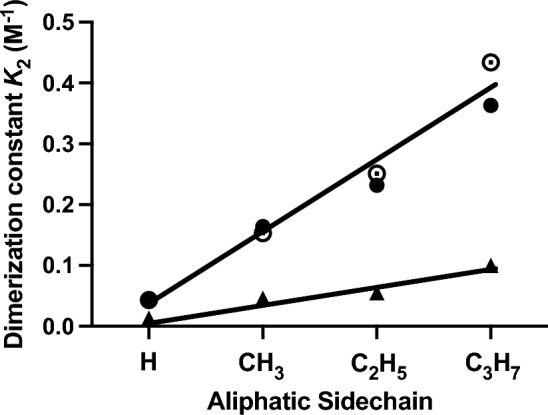
Variation of the dimerization constant for carboxylic acids (25 °C) with size of the aliphatic chain. ●, Data based on conductivity measurements taken from Table I of Katchalsky et al. (1961): ▲, Amended values after allowance for viscosity effects—taken from Table 4 of Cartwright and Monk (1955)]; ○, Data obtained from ionization measurements based on potentiometric titrations to determine log [H^+^] (Martin and Rossotti 1959) as interpreted in Table 4 of Schrier et al. (1964)

The magnitudes of dimerization constants thus obtained (Katchalsky et al. 1951) from the empirical *B* values (Saxton and Darken 1940) for formic acid, propionic acid and butyric acid as well as acetic acid (●) exhibit a systematic increase with increasing length of the aliphatic chain (Fig. 5). Allowance for an effect of solution viscosity on conductance measurements (Cartwright and Monk 1955) leads to lower estimates of $K_2$ (▲, Fig. 5) without affecting the finding that the dimerization constant for butyric acid is tenfold larger than that for formic acid, Also shown in Fig. 5 are dimerization constants obtained by potentiometric titrations, which afford quantification of the extent of ionization from the variation in pH = ‒log $C_{H^+ }$ (Martin and Rossotti 1959; Schrier et al. 1964). Those values (○) are inferred from Table III of Schrier et al. (1964), which makes allowance for medium effects at the high ionic strength (0.3 M) of the potentiometric titrations.

### Effect of temperature on the dimerization constant for small solutes

In an attempt (Schrier et al 1964) to determine the standard enthalpy of dimerization (Δ$$H^o$$) from the temperature dependence of the standard free energy (Δ$$G^o )$$, potentiometric titrations of formic acid in 0.3 M NaCl were performed at four temperatures (10, 25, 40, 55 °C). Their observation of temperature independence of ln $$K_2$$ was taken to signify a value of zero for $$\Delta H^o$$.

A similar situation had already been encountered in an investigation of the self-association of N-methylacetamide in aqueous solution (Klotz and Franzen 1962). In that early application of IR spectroscopy for the study of interactions in aqueous solution the formation of N‒H^**…**^O=C bonds was used to monitor self-association at 25 °C and 60 °C. Studies of N-methylacetamide in a nonaqueous solvent (benzene) had established the equilibrium coexistence of monomers, dimmers, trimers, etc., (Davies and Thomas 1956) for which the stepwise association constant is smallest for dimer formation. Advantage was taken (Klotz and Franzen 1962) of the fact that each oligomer also has one free-imino group to calculate $C_f$, the molar concentration of free_-_imino groups in a solution with total concentration $C$ of methylacetamide; and hence the fraction of complexed NH groups, $\alpha = (C - C_f )/C$, as well as the fraction free, $(1 - \alpha )$. Because the concentration of monomer equates with $C_f$ in the limit of zero solute concentration (*α* → 0), the dimerization constant was evaluated as the ordinate intercept of the dependence of $\alpha /(1 - \alpha )C_f$ upon *α*. Effects of thermodynamic nonideality on the magnitude of $K_2$ were also eliminated by this extrapolation to zero solute concentration. The application of this approach to results for aqueous N-methylacetamide solutions at 25 °C yielded an association constant of 0.005 M^‒1^ [Table 1 of Klotz and Franzen (1962)]; and the return of a similar estimate of $K_2$ from IR measurements 60 °C was also taken to signify a value of zero for Δ$H^o$.

These attempted interpretations without separate characterization of $\Delta H^o$ (Klotz and Franzen 1962; Schrier et al. 1964) both imply that the dimerization is entropically driven ($\Delta G^o = - T\Delta S^o )$, an observation that seemingly confirms the original concept of hydrophobic interaction as the clustering of hydrophobic groups away from the aqueous environment (Kauzman 1959). The concept of hydrophobic interactions as a source of additional free energy was invalidated subsequently (Lumry and Rajender 1970) by findings of a linear dependence between $\Delta H^o$ and $\Delta S^o$ in experimental studies of protein‒ligand interactions where the standard enthalpy and standard free-energy changes were both measured (Anusiem et al. 1968; Eftink et al. 1983; Kilpatrick et al. 1986; Edwards et al. 2009; Breiten et al. 2013; Kang and Smidtas 2021). The temperature independence of $\Delta G^o$ in the above studies of small solute dimerization (Klotz and Franzen 1962; Schrier et al. 1964) was thus more likely to be signifying enthalpy‒entropy compensation ($\Delta H^o - T\Delta S^o = 0$), particularly in light of the results for urea dimerization (Schellman 1955).

## Source of the enthalpy‒entropy compensation

**Fig. 6:**
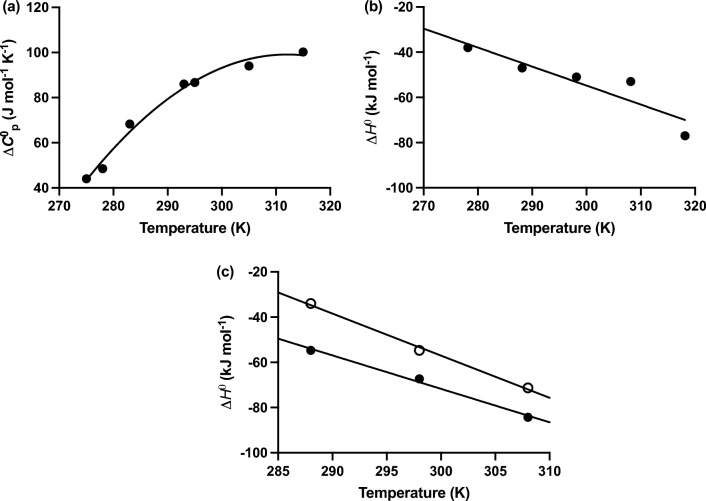
Demonstration of different roles for water involvement in enthalpy‒entropy compensation. **a** Recognition of water as a competitive inhibitor of urea dimerization from positive temperature dependence of the heat capacity Δ$C_p^o$. [Data taken from Fig. 3 of Gucker and Pickard (1940).] **b** Negative temperature dependence of Δ$H^o$ for the binding of the dipeptide AA to the dipeptide-binding protein DppA reflecting the presence of structured water molecules in the hydrophobic binding site region located in the interior of the DppA‒AA complex. [Data taken from Table 1 of Zainol et al. (2021).] **c** Temperature dependence of Δ$H^o$ for HIV-1 protease inhibition by KNI-10033 (○) and KNI-10075 (●), which differs from the former by the replacement of a thioether group by a sulphonyl counterpart (see Fig. 7). [Data taken from Fig. 4 of Lafont et al. (2007).]

The fact that account has been taken of the energetics of the whole system renders the dimerization of urea as the logical starting point in this search for the source of enthalpy‒entropy compensation in the self-association of small solutes. Some entropic disadvantage must inevitably emanate from the restricted relative movement of two urea molecules comprising a dimer. However, the large magnitude of the negative $T\Delta S^o$ contribtion to $\Delta G^o$ signifies the presence of additional sources for the observed enthalpy‒entropy compensation. Because the hydroxyl groups of water are certainly contenders for hydrogen-bond formation with the carbonyl group of the urea monomer, water molecules have the capacity to act as a competitive inhibitor of urea dimerization. That interpretation is in keeping with theoretical predictions of the chemical structure of the urea dimer (Hernandez-Cobos et al 1993; Isheda et al. 2004; Stumpe and Grubmüller 2007; Ramondo et al. 2007). It is also consistent with the positive temperature dependence of the molal heat capacity for aqueous urea presented in Fig. 6a, where the values of Δ$C_p$ have been taken as the ordinate intercepts of apparent values obtained over a range of urea concentrations (Gucker & Ayres 1937; Gucker and Pickard 1940). This positive temperature dependence of Δ$C_p$ would then imply the progressive weakening of all hydrogen bonds (urea‒urea as well as urea‒water) as required by the van’t Hoff isochore for an enthalpically driven interaction (Δ$H^o$ negative). In retrospect those early studies of the energetics of aqueous urea solutions provided the first warning of the need for caution in regarding water as an inert solvent.

Temperature-independence of the dimerization constants for formic acid (Schrier et al. 1964) and N-methylacetamide (Klotz and Franzen 1962) is also consistent with the concept of water involvement in the energetics of the systems. Indeed, competition from water hydroxyls for the carbonyl group involved in dimer formation provides a highly plausible explanation of the relatively small extent of dimerization ($K_2$ = 0.03 M^‒1^) observed for formic acid at 25 °C. Furthermore, the placement of water near a hydrophobic environment gives rise to an enhanced enthalpic contribution to Δ$G^o$ (and $K_2$) for successive aliphatic acids (Fig. 5) by enhancing the competition of water hydroxyls for the solute carbonyls because of the greater strength of water structure (hydrogen bonding) in an increasingly hydrophobic environment (Kauzman 1959; Tanford 1980). That explanation would also account for the much smaller magnitude of $K_2$ for N-methylacetamide.

## Enthalpy‒entropy compensation in protein‒ligand interactions

In studies of ligand interactions with macromolecular acceptors attention has been accorded an alternative means by which water can contribute to the energetics of reactions in aqueous solution—its adoption of a more rigid structure in a hydrophobic environment (Kauzman 1959; Tanford 1980). Under circumstances where the predominant source of enthalpy‒entropy compensation is the strengthening of water structure in a hydrophobic environment, the corresponding temperature dependence of Δ$C_p$ is negative (Eftink et al. 1983)—a feature also shown in Fig. 6b for the interaction of the alanine dipeptide AA with DppA, the dipeptide-binding protein that facilitates their transport through the cytoplasmic membrane (Zainol et al. 2021). Although the strength of hydrogen bonding still decreases with increasing temperature, the opportunity for further strengthening of water structure effected by the hydrophobic environment is minimal at low *T* because the strength of water‒water hydrogen bonds is already approaching maximal. The inverse temperature dependence of Δ$C_p$ in Fig. 6b is thus reflecting the enhanced randomness of water structure at higher temperature and hence a greater capacity for the adoption of a more rigid water structure (Zainol et al. 2021). The existence of this potential source of enthalpy‒entropy compensation is evident from the X-ray crystallographic structures of several periplasmic binding proteins, where the attachment of ligand to the binding site results in its encapsulation with a number of structured water molecules in a hydrophobic region of the protein with no access to the aqueous environment (Quiocho 1990; Tame et al. 1994; Dunten and Mowbray 1995).

**Fig. 7:**
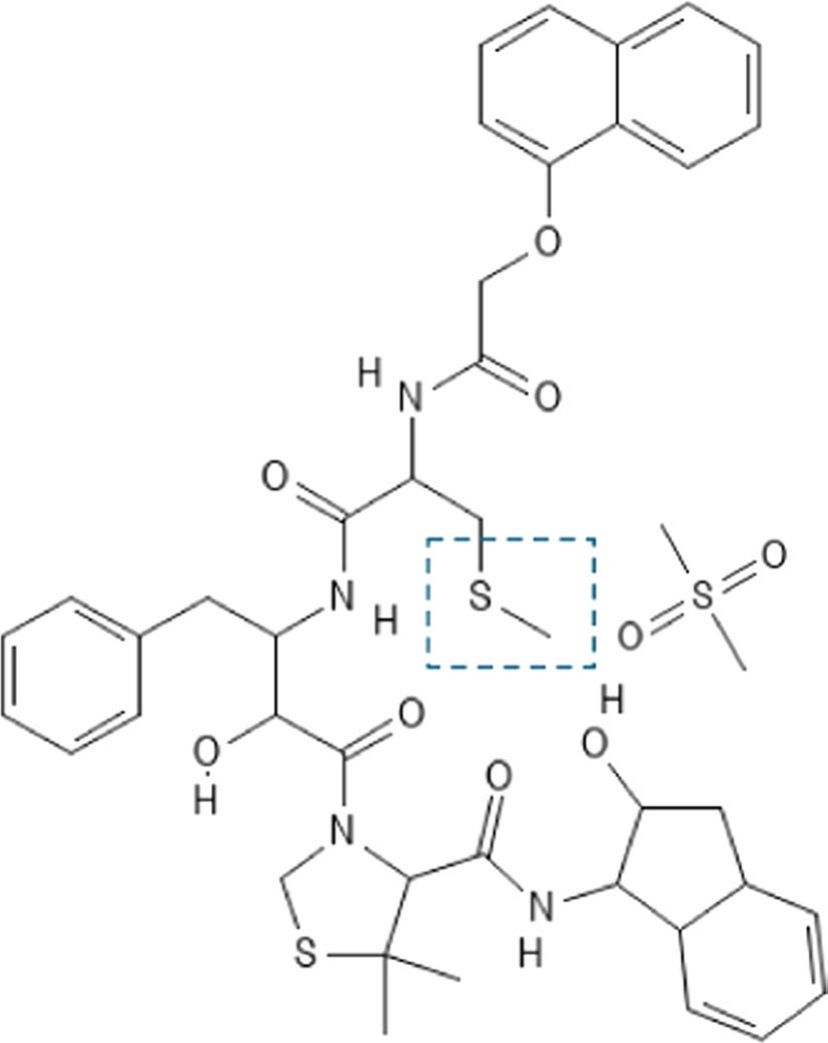
The chemical structure of HIV-1 protease inhibitor KNI-10033, together with the change made at the indicated position to generate KNI-10075, an inhibitor with two additional hydrogen-bonding groups as the result of the sulphonyl/thioether substitution

The source of enthalpy‒entropy compensation arising from water involvement in small-solute dimerization thus differs from that mainly responsible for the phenomenon in interactions of ligands with macromolecular acceptors. However, the potential for water acting as a competitive inhibitor to protein‒ligand interaction certainly exists; and may well have contributed to the thwarted attempt (Lafont et al. 2007) to strengthen the inhibition of HIV-1 protease by the introduction of extra sulfonyl counterparts (‒S=O) into an already powerful inhibitor (K = 8.3 × 10^10^ M^‒1^). The interaction of that unmodified-inhibitor molecule, KNI-10033 (Fig. 7), with HIV-1 protease provides an example of the situation in which the enthalpic and entropic contributions to the energetics of complex formation are both favorable: Δ$$G^o = - 62.3 $$ kJ/mol, Δ$$H^o = -$$34.3 kJ/mol, Δ$$TS^o$$ =  + 27.9 kJ/mol (Lafont et al. 2007). Similar findings of extreme enthalpy‒entropy compensation have been reported for the binding of inhibitors to a drug-resistant variant of HIV-1 protease (King et al. 2012). In aqueous solution structured water covers the ligand as well as the protease active site because of their hydrophobicity. Complex formation between ligand and the protease active site region thus involves the concomitant displacement of this structured water into the aqueous environment in the process termed cavity desolvation. Because this structured water release includes contributions from the ligand as well as the protein acceptor for the present system, the entropic energy gain is even greater than that encountered in systems such as oligopeptide binding to OppA (Tame et al. 1994), protein‒DNA interactions (Privalov et al. 2007, 2011; Dragan et al. 2017) and the broad ligand binding selectivity for rat odorant binding protein 3 (Portman et al. 2014).

In an attempt to increase inhibitor potency the thioether residue (‒SCH_3_) at the indicated position in KNI-10033 (Fig. 7) was replaced by its sulfonyl counterpart (‒SO_2_CH_3_) to introduce two additional hydrogen-bonding groups, one of which formed an extra bond with the peptide imino at D30 on the B-chain of HIV-1 protease. Isothermal titration calorimetry of this modified system revealed an enthalpic energy gain of 16.4 kJ/mol (Δ$H^o$ = ‒50.7 cf ‒34.3 kJ/mol) but an entropic loss of 17.4 kJ/mol (TΔ$S^o$ =  +10.5 cf + 27.9 kJ/mol), leaving the standard free energy essentially unchanged (Δ$G^o$ = ‒61.1 cf ‒62.3 kJ/mol). As well as reflecting the consequences of structured water within the hydrophobic environment of the buried protease active-site region, this example of enthalpy‒entropy compensation could well incorporate contributions from direct water involvement via hydrogen bonding to the additional ligand sulfonyl groups—the analogous interaction responsible for the phenomenon in small-solute dimerization. In that regard the unliganded inhibitor KNI-10075 would contribute a higher enthalpic contribution than KNI-10033 by virtue of such binding of water to both sulfonyl groups that would be countered by a corresponding loss in its entropic counterpart through decreased randomness of water structure. Involvement of one of these two KNI-10075 carbonyl counterparts in hydrogen-bond formation with the backbone-peptide imino at D30 in the protease-B chain would then be of little energetic advantage because its creation is at the expense of the existing hydrogen bond with water. On the other hand, the enhanced enthalpic and decreased *T*Δ$S^o$ energetic contributions arising from hydrogen bonding between water and the second ligand sulfonyl would still be part of the overall energetics of the system; and thus account for the compensating changes observed in enthalpic and entropic inputs into the standard free energy for protease-complex formation with the two ligands. Further support for that contention comes from a comparison of the temperature dependence of Δ$H^o$ for the two systems shown in Fig. 6c. Although the slopes for both interactions signify a negative heat capacity change that is consistent with findings for other protein‒ligand interactions, the smaller magnitude of that overall negative Δ$C_p$ for the interaction with modified ligand KNI-10075 (●) can be rationalized in terms of a superimposed positive heat capacity input stemming from the hydrogen-bond formation between water and ligand seen in studies of urea dimerization (Fig. 6a).

## Concluding remarks

Despite the surprise and criticism generated by reports of the existence of extreme enthalpy‒entropy compensation in protein‒ligand interactions (Gilli et al. 1994; Sharp 2001; Cornish-Bowden 2002; Krishnamurthy et al. 2006), the phenomenon had been observed much earlier in the context of urea self-association (Schellman 1955). An important factor in its re-emergence in studies of protein‒ligand interactions has been the development of isothermal titration calorimetry (Wiseman et al. 1989) for the concurrent-independent estimation of Δ*H*^*o*^ and Δ$G^o$ (via the equilibrium constant)—a development that has helped to counter early considerations of the phenomenon as a potential statistical artifact (Sharp 2001; Cornish-Bowden 2002). A procedure is now in place (Griessen and Dam 2021) to make allowance for the consequences of statistical uncertainty, which continue to be raised (Chodera and Mobley 2013).

In the absence of information about the magnitude of Δ$H^o$ the observation of a temperature-independent equilibrium constant can be misconstrued as signifying a value of zero for Δ*H*^*o*^—the situation almost certainly encountered in studies of the self-association of N-methylacetamide (Klotz and Franzen 1962) and formic acid (Schrier et al. 1964) by virtue of analyses based on assumed inertness of the solvent (water). In retrospect, the problems encountered in the search for a satisfactory molecular explanation of enthalpy‒entropy compensation for so long have been largely self-inflicted by a reluctance of researchers to include the solvent as part of the thermodynamic system under investigation.

Because of the many ways in which water can contribute to enthalpy‒entropy compensation in protein‒ligand interactions, there is no general treatment that can be applied to predict its consequences in any given system: Complementary procedures such as X-ray crystallography and NMR can certainly be used to deduce the existence of an entropic energy gain effected by cavity desolvation (Portman et al. 2014) or of the enthalpic energy gain arising from entrapment of water molecules within a highly hydrophobic environment (Quiocho 1990; Tame et al. 1994; Dunten and Mowbray 1995). However, the compensation derived from the smaller extents of solvent-structure strengthening in the aqueous environment adjacent to exposed hydrophobic residues poses a challenge to experimental detection. Although computational procedures sufficed to establish the structural role of water in the interactions (solvent‒solute, solvent‒ligand, solvent‒solvent) as the major source of extreme enthalpy‒entropy dimerization of urea (Stumpe et al. 2007; Ramondo et al. 2007), their use to define the sources of enthalpy‒entropy compensation in biomolecular recognition (Peccati and Jiménez-Osés 2021) poses far greater problems because of the variety of ways in which water can affect the phenomenon. Nevertheless, the consideration of solvent compensation has the obvious advantage that any favorable enthalpic contribution to $\Delta G^o$ from enhanced hydrogen bonding involving water molecules is necessarily offset by a concomitant loss because of decreased randomness of a more ordered water structure. Conversely, any favorable entropic contribution to $\Delta G^o$ from cavity desolvation gives rise to a decreased enthalpic contribution because of the hydrogen-bond rupture associated with generation of the more random water structure. Extreme enthalpy‒entropy compensation in protein‒ligand systems is thus an inbuilt part of any interaction involving the solvent because the enthalpic and entropic contributions to $\Delta G^o$ self-cancel.
